# Development of a Vertical Submerging and Emerging Bat-Ray-Inspired Underwater Vehicle

**DOI:** 10.3390/biomimetics9100582

**Published:** 2024-09-25

**Authors:** Enrique Mar-Castro, Sergio Alejandro May-Rodríguez, Rafael Stanley Núñez-Cruz, Elba Dolores Antonio-Yañez, Luis Mario Aparicio-Lastiri, Juan Herrera-Vidal

**Affiliations:** Control and Design Laboratory, Polytechnic University of Tulancingo, Tulancingo de Bravo 43629, Mexico; enrique.mar2415005@upt.edu.mx (E.M.-C.); sergio.alejandro2231092@upt.edu.mx (S.A.M.-R.); elba.antonio@upt.edu.mx (E.D.A.-Y.); luis.aparicio2415004@upt.edu.mx (L.M.A.-L.); juan.herrera2315007@upt.edu.mx (J.H.-V.)

**Keywords:** bio-inspired design, remote-operated vehicle, mathematical modeling of underwater robots, vertical submerging and emerging underwater vehicles

## Abstract

In this article, the development of a bat-ray-inspired underwater vehicle is presented; although the propulsion of the vehicle is based on traditional thrusters, the shape of the ray’s fins was used as a model to design the body of the vehicle; this architecture allows the independent control of the forward velocity and the full attitude of the vehicle using only two thrusters and two articulated fins. The compact design of the robot, along with the high dexterity of the architecture, allows the vehicle to submerge and emerge vertically as well as navigate horizontally. The mathematical model of the proposed vehicle, including dynamics and propulsion system, is presented and validated using numerical simulations. Finally, experimental tests are presented to demonstrate the capabilities of the proposed design.

## 1. Introduction

Across the vast expanse of the oceans, researchers and engineers have been inspired by the complex designs and amazing abilities of nature’s aquatic inhabitants, creating a new generation of bio-inspired underwater vehicles. Bio-inspired machines seek to achieve the unique capabilities that allow aquatic animals to move efficiently, live under high atmospheric pressures, and conserve energy via mimicking the morphology, behavior, and locomotion of these organisms.

Biomimetics of marine animals has been applied to the design of sensors, locomotion mechanisms, and to define the shape of robot bodies. Some applications of bio-inspired sensors are the measurement of the velocity of fluid surrounding a robot [1], detection of disturbances in fluids [2], and emulating touch and obtaining information about objects grabbed by a manipulator [3], among others.

Locomotion inspired by marine animals focuses on replicating the efficient movement mechanisms and swimming patterns of creatures such as fish, cephalopods, and aquatic mammals by using fins, tails, and flexible structures. According to Sun et al. [4], there are two basic forms of locomotion: the first is known as body and caudal fin (BCF), in this type of swimming the body is bent in a wave-backward propulsion that extends to the tail fin. The second form of basic locomotion is the so-called median and paired fin (MCF), in which the swimmer uses these elements to gain propulsion. Currently, different robots have been developed that imitate these types of locomotion; for example, fish-based robots [5,6] can use a system of pectoral and caudal fins to maneuver in small spaces and perform rapid turns, while those inspired by cephalopods [7] can take advantage of jet propulsion to achieve fast and precise movements.

The morphology of aquatic animals provides them with characteristics and hydrodynamic capabilities that are of great interest for the development of new robotic prototypes. For example, robots that emulate sea snakes [8,9] use flexible, segmented bodies to move nimbly in complex underwater environments, replicating their undulating movements. Jellyfish [10,11], with their jet propulsion system based on the contraction and expansion of their bell, have inspired the design of robots that can navigate smoothly and with energy consumption reduced. Turtles [12,13], with their ability to navigate long distances and their efficient hydrodynamic profile, are also being studied to improve the navigability and efficiency of underwater robots in long-distance explorations and in diverse marine environments. Finally, the rays [14,15,16], with their undulating flapping motion, have served as a model for developing robotic fins that provide superior maneuverability in underwater robots, in addition to their structure providing greater stability.

To select the marine animal intended for mimicry, it is necessary to carry out an analysis of the project’s needs. Some variables to consider are maneuverability, flexibility, hydrodynamics, speed, and stability of the platform. In this work, a platform is sought that has high maneuverability and good hydrodynamic characteristics, which allow inspection and marine archaeology tasks to be carried out. With this in mind, and having made a comparison of the different existing structures, the one based on a ray was selected. These animals have high maneuverability and stability; however, their movement is slow and inefficient and replicating the movement of the fins is a very complicated task due to the large number of muscles that these animals possess. For this reason, the maneuverability that many of these prototypes have is classified between low and medium.

The aim of this project was to develop a novel unmanned underwater vehicle which is stable, fast, maneuverable, able to navigate efficiently, but also capable of hovering over a specific area for inspection or mapping applications. To accomplish this goal, the authors propose a novel design which, although it is actuated using traditional thrusters, is able to exhibit high moving efficiency because the shape of the body is inspired by the shape of the ray’s fins, which are articulated to produce changes in the direction of movement.

**Figure 1:**
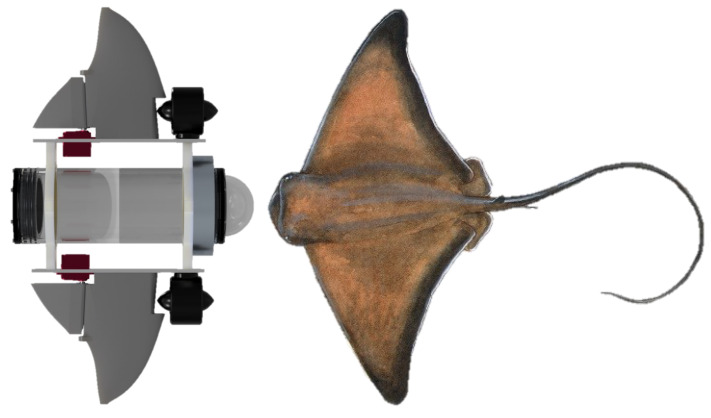
On the left side is the proposed vehicle, on the right side a photo of a bat ray; extracted from the “Marine Species Portal” at https://marinespecies.wildlife.ca.gov/bat-ray/, accessed on 19 August 2024.

Figure 1 shows the exterior design of the proposed vehicle, although there are benefits in trying to fully mimic the shape and locomotion of bat rays, the resulting designs are usually bulky and not sufficiently agile [17]. On the other hand, traditional propulsion technologies are well developed and easy to control for high-speed underwater locomotion. In the proposed design, the downside of using thrusters as the propulsion system is compensated by the hydrodynamics of the ray’s shape to produce a design with high maneuverability, agility, and efficiency characteristics.

## 2. Bio-Inspired Robot Design

The design of an underwater robot involves many important choices, for example, the type of shape, hull, and locomotion, etc. That is why several aspects need to be considered like the pressure, work depth, versatility, and size requirements. In this regard, bio-inspiration is a good option in robot design because marine animals can serve as benchmarks with respect to different characteristics like swimming speed, efficiency, maneuverability, and stealth [18].

### 2.1. Speed

Bio-inspiration in locomotion or morphology seeks to achieve the speed of marine animals because they are, aside from some exceptions, faster than traditional AUVs both in terms of absolute velocity and relative velocity (the speed normalized with respect to their body length (BL)) because of their swimming system and the drag reduction of their bodies, developed by years of underwater evolution [18,19].

### 2.2. Efficiency

Efficiency pertains to power usage and determines the distance that can be covered and the operational duration for a specific amount of fuel or energy [18]. It can be measured with the cost of transport (*COT*), which quantifies the energy expenditure required to swim at a given speed and is inversely proportional to efficiency [20,21]. Equation (1) shows the mathematical expression of this parameter [22]:

(1) $$COT≜\frac{E}{mgd}=\frac{P}{mgv}$$

where *E* and *P* are the energy and power of the system, *d* and *v* are the distance traveled and the velocity of the vehicle, *m* is the mass of the system, and *g* is the acceleration due to gravity, for the units of measurement of the mentioned variables, the International System of Units is used.

### 2.3. Maneuverability and Agility

Maneuverability is the capability to change speed, direction, and location while maintaining stability and can be described as the capability to turn in a confined space; it involves fast and stable navigation even at low speeds. Maneuvering can be focused on lateral turning; that is why maximal maneuverability is usually associated with the minimum turn radius; with this definition it can be measured as the length-specific radius $r_{ls}$ of the turn trajectory [23,24].

The agility is the rate of turn while changing directions and is measured as the change in angular velocity [25]. The concept of agility also includes the concept of maneuverability, but in this case speed agility is the ability of the vehicle to accelerate or decelerate [23].

### 2.4. Bio-Inspired Rajiform Underwater Robot

When talking about rajiform bio-inspiration, the most common approach is to reproduce their locomotion by trying to replicate the pectoral fin system using multiples actuators; there are some examples of this approach, like [15], where the robot has fourteen degrees of freedom, using three servomotors on each side of the fin design and with three ribs attached to the axis. In [26], the robot has pectoral fins that consist of three complex mechanisms with the goal of resembling the skeleton of the real fins. In [27], the design consists of five motors on each fin and two propellers located in the body of the robot.

The downside of these designs is the fact that the used fins are not as flexible as those of fish, resulting in lower degrees of freedom and loss of maneuverability [4,28] and the fact that multiple actuators positioned within the fins in this kind of robot may present restrictions in the design and sizing of the system, in addition to greater energy consumption.

Another approach [15] consists of modeling the oscillatory movement of the fin through the combination of a synchronized flapping movement and a pitching rotation of the fin. In [15,29,30], the strategy used to model rajiform locomotion is the use of flexible materials to build the fin and a single actuator to move each one of them; this translates to a degree of freedom in each one of the fins; the undulation then is obtained passively. The disadvantages of these robots is that the maneuverability is decreased.

As can be seen in the prototypes mentioned above, the efforts to imitate the form of locomotion of rajiforms made them use a large number of actuators, which make the systems very complex, and therefore, important characteristics such as speediness or maneuverability are sacrificed. For this reason, the inspiration for this work was mainly based on the hydrodynamic advantages of the shape of bat rays.

According to the previously mentioned literature, bat rays exhibit lower drag and high stability, characteristics that are highly desirable for an underwater vehicle. One way to reproduce the contour of the fin is to approximate it with a series of aerodynamic profiles with gradient changes from a large profile in the center of the body to a small profile at the tip of the fin [31]. The thickness of the body should follow the dimensions of the body and the body should be flattened [32].

**Figure 2:**
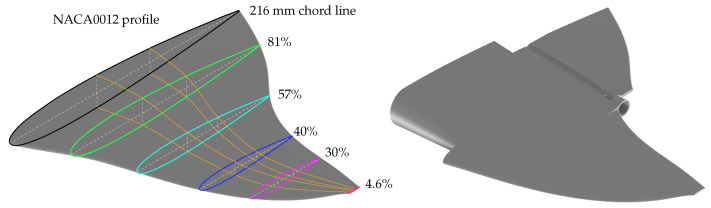
Design of the robot fins based on the NACA0012 profile.

In the proposed design, a series of scaled NACA0012 profiles were used and a joint was added to redirect the flow through the fins, as shown in Figure 2. In this part of the design, the Fusion 360 v2.0 software was used, along with two guide profiles. The smallest chord line, located at the end of the fin, measures 10 mm, while the longest is 216 mm.

**Figure 3:**
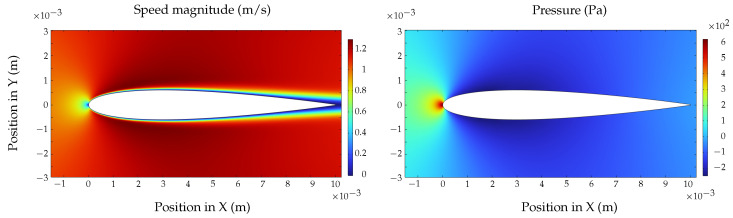
Simulation in Comsol Multiphysics 6.2 to observe the behavior of surrounding fluid around profile NACA0012.

The NACA0012 profile allows us to simplify the vehicle model as it is a symmetrical profile. In Figure 3, two simulations can be observed, carried out in Comsol Multiphysics, that show the behavior of the profile when a water flow at 1 m/s passes around its geometry. Since it is a symmetrical profile, the speed of the fluid passing above it is the same as that passing below it, this is also the case for pressure. It is worth mentioning that this simulation was carried out for an angle of attack of $0^{\circ }$, generating a cancellation in the lift forces.

The locomotion of our prototype is based on two thrusters and two control surfaces (fin tips) manipulated by a servomotor in each one. Because the propeller is located in front of the fin, it is possible to modify the direction of the fluid through the control surfaces.

**Figure 4:**
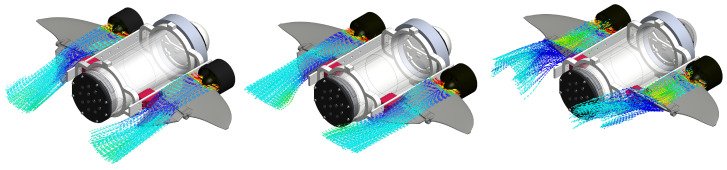
Simulation of the modification of fluid flow direction around the fin through the action of control surfaces.

Figure 4 shows three general cases of how the flow is redirected to modify the motion of the vehicle. These simulations were carried out using the Flow Simulation plugin of the SolidWorks 2023 software. This mechanism allows different forces and torques to be generated, as shown in the vehicle model section.

## 3. Modeling, Simulation, and Control

**Figure 5:**
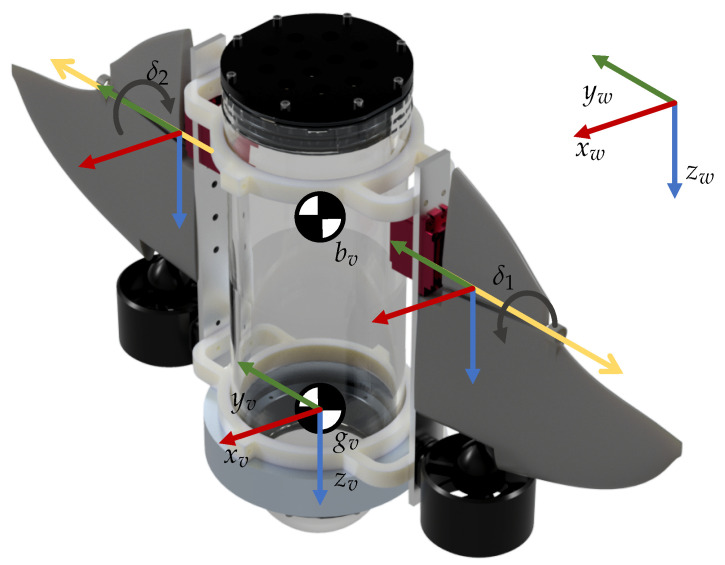
Locations of the coordinate systems for the dynamic analysis.

The platform proposed in the previous section is characterized by having two thrusters and two fins that are placed symmetrically, which we refer to with the index i∈{1,2}, where 1,2 represent the left and the right sides, respectively. Figure 5 shows the structure of the platform and the coordinate systems used.

In Figure 5, the unitary vectors $x_{w}$, $y_{w}$, and $z_{w}$ form the coordinate system of the world $O_{w}$, similarly $x_{v}$, $y_{v}$, and $z_{v}$ form the coordinate system of the vehicle $O_{v}$, the center of gravity (CoG) of the vehicle is denoted as $g_{v}$ and it is located at the origin of $O_{v}$, the center of buoyancy (CoB) of the vehicle is denoted as $b_{v}$, and the angle of the fins is represented by $\delta_{i}$.

To define the mathematical model of the system, a simplified notation is defined for position *r*, linear velocity *v*, angular velocity ω, linear acceleration *a*, and angular acceleration α; this notation uses only one subscript, i.e., $r_{i}$ denotes the position of origin $O_{i}$ relative to the world frame $O_{w}$ expressed in local frame $O_{i}$. An explicit notation is defined for the general case, this notation uses two subscripts j/i, which indicate the related frames, and one superscript *k*, showing the frame used to express the quantity, i.e., $r_{j/i}^{k}$ denotes the relative position of origin $O_{j}$ relative to frame $O_{i}$ expressed in frame $O_{k}$.

The notation for rotations is defined as $R_{j/i}$, which denotes the rotation matrix of origin $O_{j}$ relative to $O_{i}$, then it can be used to transform vectors in $O_{i}$ to $O_{j}$.

### 3.1. Mathematical Model

The dynamic model of the UAV can be obtained by applying the Newton–Euler methodology through an analysis of the forces and torques acting on the vehicle. In this work, the entire structure has been considered as a particle because the weight of the fin tips, compared to the mass of the vehicle, is very small.

The relation between the total force $F_{v}$ and moment $N_{v}$ applied to the vehicle’s CoG and the movement generated can be expressed in frame $O_{v}$, as defined in Equation (2):

(2a) $$\begin{array}{cc} F_{v} & =m_{v}a_{v} \end{array}$$

(2b) $$\begin{array}{cc} N_{v} & =I_{v}\alpha_{v}+\omega_{v}\times \left(I_{v}\omega_{v}\right) \end{array}$$

where $m_{v}$ and $I_{v}$ represent the mass and inertia matrix of the vehicle, measured at the CoG of the vehicle [33].

The total force $F_{v}$ and moment $N_{v}$ are calculated in terms of the external forces of the environment and actuation forces of the vehicle, as defined in Equation (3):

(3a) $$\begin{array}{cc} F_{v} & =f_{g}+f_{b}+f_{a}+f_{d}+f_{p} \end{array}$$

(3b) $$\begin{array}{cc} N_{v} & =n_{g}+n_{b}+n_{a}+n_{d}+n_{p} \end{array}$$

where $f_{g}$ and $n_{g}$ represent the force and moment produced by gravity on the vehicle; $f_{b}$ and $n_{b}$ represent the force and moment produced by the buoyancy of the vehicle; $f_{a}$ and $n_{a}$ represent the force and moment produced by the added mass of the vehicle; $f_{d}$ and $n_{d}$ represent the force and moment produced by the drag of the vehicle; and $f_{p}$ and $n_{p}$ represent the force and moment produced by the propeller thrusts of the vehicle. All forces and moments are defined relative to frame $O_{v}$.

Each one of the elements in Equation (3) is defined in the next subsections.

#### 3.1.1. Gravitational Forces

The gravitational force of the vehicle $f_{g}$, expressed in $O_{v}$, is defined in Equation (4); $f_{g}$ does not produce a moment because it is calculated at the vehicle’s CoG:

(4a) $$\begin{array}{cc} f_{g} & =m_{v}gR_{v/w}z_{w} \end{array}$$

(4b) $$\begin{array}{cc} n_{g} & =0 \end{array}$$

where g is the acceleration due to gravity.

#### 3.1.2. Buoyancy Forces

The buoyancy force $f_{b}$ is proportional to the mass of the fluid displaced by a moving body, in the opposite direction to the gravitational force [34]; by Archimedes’ principle it is defined as follows in Equation (5):

(5a) $$\begin{array}{cc} f_{b} & =-m_{f_{v}}gR_{v/w}z_{w} \end{array}$$

(5b) $$\begin{array}{cc} n_{b} & =r_{b_{v}/v}^{v}\times f_{b} \end{array}$$

where $m_{f_{v}}$ is the mass of the fluid displaced by the vehicle, calculated as $m_{f_{v}}=\rho_{fl}\nabla_{v}$, where $\rho_{fl}$ is the density of the fluid and $\nabla_{v}$ the volume of the fluid displaced by the vehicle. The position of the center of bouyancy relative to $O_{v}$ is denoted as $r_{b_{v}/v}^{v}$.

#### 3.1.3. Added Mass Forces

When a submerged body moves, it must displace a volume of the fluid that surrounds it. In the hydrodynamics field, this phenomenon can be modeled as a virtual mass added to the system [35].

The mathematical expression of added mass forces highly depends on the geometry, velocity of the vehicle, frequency of the fluid, etc.; when considering a symmetric body and irrotational ocean currents, it can be approximated as shown in Equation (6):

(6a) $$\begin{array}{cc} f_{a} & =-m_{f_{v}}a_{vr} \end{array}$$

(6b) $$\begin{array}{cc} n_{a} & =-I_{a_{v}}\alpha_{v}-\omega_{v}\times \left(I_{a_{v}}\omega_{v}\right) \end{array}$$

where $I_{a_{v}}$ is an inertia matrix due to the added vehicle mass and $a_{vr}$ is the acceleration of the vehicle relative to the surrounding fluid, defined as $a_{vr}=a_{v}-R_{v/w}a_{fl}$, where $a_{fl}$ is the acceleration of the fluid expressed in $O_{w}$. Considering an irrotational fluid implies $\omega_{fl}=0$.

#### 3.1.4. Damping Forces

Another hydrodynamic effect is the damping caused by the fluid’s viscosity that causes dissipative forces of drag (profile and superficial friction) and lifts that act on the body’s center [36]. The lift forces are orthogonal to the velocity of the fluid, and the drag forces are parallel to the velocity of the fluid and act on the CoM of the body [37].

Damping forces and moments are nonlinear and coupled; the following Equation (7) represents only the linear decoupled part of these phenomena:

(7a) $$\begin{array}{cc} f_{d} & =-d_{v}v_{vr} \end{array}$$

(7b) $$\begin{array}{cc} n_{d} & =-D_{v}\omega_{v} \end{array}$$

where $D_{v}$ and $d_{v}$ represent the linear coefficients of the damping forces.

#### 3.1.5. Propeller Forces

The propeller force $f_{p_{i}}$ is defined as shown in Equation (8):

(8a) $$\begin{array}{cc} f_{p_{i}} & =f_{t_{i}}R_{y,\delta_{i}}z_{v} \end{array}$$

(8b) $$\begin{array}{cc} n_{p_{i}} & =r_{p_{i}/v}^{v}\times f_{p_{i}} \end{array}$$

where $f_{t_{i}}$ is the force generated by thruster $t_{i}$ and $R_{y,\delta_{i}}$ is the rotation matrix around the *y*-axis that defines the orientation of fin *i*, with a rotation $\delta_{i}$ (rad). The position where $f_{p_{i}}$ is applied to the vehicle, relative to $O_{v}$, is denoted as $r_{p_{i}/v}^{v}$.

### 3.2. Simulation

Using Equations (2)–(8), the dynamic model can be solved to obtain the acceleration vector relative to $O_{v}$, denoted by $\dot{\nu }={\left[\begin{array}{cc} a_{v} & \alpha_{v} \end{array}\right]}^{⊺}$; the numerical integration of $\dot{\nu }$ is used to calculate the velocity vector $\nu ={\left[\begin{array}{cc} v_{v} & \omega_{v} \end{array}\right]}^{⊺}$; however, the integration of ν has no physical sense [35].

The pose of the vehicle relative to $O_{w}$ is denoted by $\eta ={\left[\begin{array}{cc} \eta_{1} & \eta_{2} \end{array}\right]}^{⊺}$, where $\eta_{1}={\left[\begin{array}{ccc} x_{v} & y_{v} & z_{v} \end{array}\right]}^{⊺}$ is the position and $\eta_{2}={\left[\begin{array}{ccc} \phi_{v} & \theta_{v} & \psi_{v} \end{array}\right]}^{⊺}$ is the attitude.

The relation between $\dot{\eta }$ and ν is expressed as shown in Equation (9) [35]:

(9) $$\left[\begin{array}{c} \dot{\eta_{1}}\\ \dot{\eta_{2}} \end{array}\right]=J\left(\eta_{2}\right)\left[\begin{array}{c} v_{v}\\ \omega_{v} \end{array}\right]=\left[\begin{array}{cc} J_{1}\left(\eta_{2}\right) & 0_{3\times 3}\\ 0_{4\times 3} & J_{2}\left(\eta_{2}\right) \end{array}\right]\left[\begin{array}{c} v_{v}\\ \omega_{v} \end{array}\right]$$

where $J_{1}\left(\nu_{2}\right)=R_{w/v}$ and $J_{2}\left(\nu_{2}\right)$ is defined depending on the rotation sequence used to define the attitude.

Transformation matrix $J(\nu_{2})$ has singularities, which depends on the chosen rotation sequence [38]; this inconvenience can be avoided by keeping the vehicle around a safe configuration; however, in this project this is not an option because it is desired that the vehicle can submerge and emerge vertically, and then, navigate horizontally to take advantage of the bio-inspired morphology.

An alternative relation to the one presented in Equation (9) is found by expressing the attitude using quaternions, which is shown in Equation (10):

(10) $$\left[\begin{array}{c} \dot{\eta_{1}}\\ {\dot{\eta }}_{2,q} \end{array}\right]=E\left(\eta_{2,q}\right)\left[\begin{array}{c} v_{v}\\ \omega_{v} \end{array}\right]=\left[\begin{array}{cc} E_{1}\left(\eta_{2,q}\right) & 0_{3\times 3}\\ 0_{3\times 3} & E_{2}\left(\eta_{2,q}\right) \end{array}\right]\left[\begin{array}{c} v_{v}\\ \omega_{v} \end{array}\right]$$

where $\eta_{2,q}={\left[\begin{array}{cccc} \epsilon_{1} & \epsilon_{2} & \epsilon_{3} & \eta_{q} \end{array}\right]}^{⊺}$ denotes the attitude expressed in the quaternion representation, which is obtained using the rotation sequence “YXZ”.

The transformation matrices $E_{1}\left(\eta_{2,q}\right)$ and $E_{2}\left(\eta_{2,q}\right)$ are defined as shown in Equation (11):

(11a) $$\begin{array}{cc} E_{1}\left(\eta_{2,q}\right) & =\left[\begin{array}{ccc} 1-2(\epsilon_{2}^{2}+\epsilon_{3}^{2}) & 2(\epsilon_{1}\epsilon_{2}-\epsilon_{3}\eta_{q}) & 2(\epsilon_{1}\epsilon_{3}+\epsilon_{2}\eta_{q})\\ 2(\epsilon_{1}\epsilon_{2}+\epsilon_{3}\eta_{q}) & 1-2(\epsilon_{1}^{2}+\epsilon_{3}^{2}) & 2(\epsilon_{2}\epsilon_{3}-\epsilon_{1}\eta_{q})\\ 2(\epsilon_{1}\epsilon_{3}-\epsilon_{2}\eta_{q}) & 2(\epsilon_{2}\epsilon_{3}+\epsilon_{1}\eta_{q}) & 1-2(\epsilon_{1}^{2}+\epsilon_{2}^{2}) \end{array}\right] \end{array}$$

(11b) $$\begin{array}{cc} E_{2}\left(\eta_{2,q}\right) & =\frac{1}{2}\left[\begin{array}{ccc} \eta_{q} & -\epsilon_{3} & \epsilon_{2}\\ \epsilon_{3} & \eta_{q} & -\epsilon_{1}\\ -\epsilon_{2} & \epsilon_{1} & \eta_{q}\\ -\epsilon_{1} & -\epsilon_{2} & -\epsilon_{3} \end{array}\right] \end{array}$$

The attitude $\eta_{2,q}$ can be obtained by numerical integration, and then, converted to Euler angles to obtain $\eta_{2}$; this approach is helpful because $E_{1}\left(\eta_{2,q}^{-1}\right)$ is used instead of $R_{v/w}$ in the equations of motion, but $\eta_{2}$ is needed to define the tracking error.

**Figure 6:**
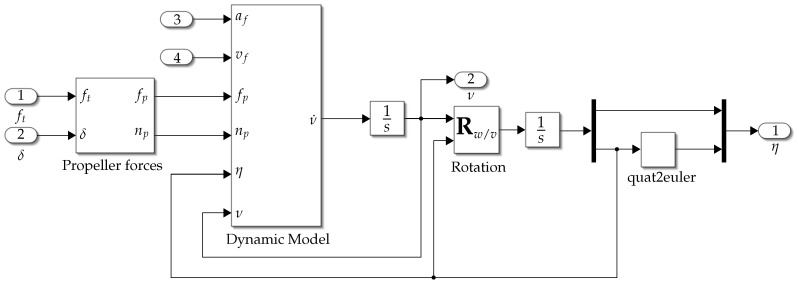
Numerical integration of the proposed mathematical model.

The procedure described in this subsection is summarized in the block diagram shown in Figure 6.

### 3.3. Control

The control problem is defined as a simultaneous forward velocity- and attitude-tracking problem. The proposed control law is a classical proportional–integral (PI) scheme, as shown in Equation (12):

(12) $$C=\left[\begin{array}{c} f_{p,z}\\ n_{p,x}\\ n_{p,y}\\ n_{p,z} \end{array}\right]=k_{p}\left[\begin{array}{c} v_{d,z}-v_{v,z}\\ \phi_{d}-\phi_{v}\\ \theta_{d}-\theta_{v}\\ \psi_{d}-\psi_{v} \end{array}\right]+k_{i}\int \left[\begin{array}{c} v_{d,z}-v_{v,z}\\ \phi_{d}-\phi_{v}\\ \theta_{d}-\theta_{v}\\ \psi_{d}-\psi_{v} \end{array}\right]dt$$

note that $f_{p,x}$ and $f_{p,y}$ are not part of control vector *C*.

If Equation (8) calculates $f_{p}$ and $n_{p}$ as a function of $f_{t_{i}}$ and $\delta_{i}$, then it is called a direct actuation model (DAM). When implementing control signals *C* as shown in Equation (12), an inverse actuation model (IAM) is needed to define actuation references given desired forces and moments.

The calculation of IAM is not straightforward because Equation (8) is nonlinear; the explicit equations for the proposed platform can be obtained by considering $r_{p_{i}/g_{v}}^{v}={\left[0,\;\mp r_{p,y},\;-r_{p,z}\right]}^{⊺}$ for i=1,2, as shown in Equation (13):

(13a) $$f_{p}=f_{p_{1}}+f_{p_{2}}=\left[\begin{array}{c} f_{p,x}\\ f_{p,y}\\ f_{p,z} \end{array}\right]=\left[\begin{array}{ccc} f_{t_{1}}\sin\delta_{1} & + & f_{t_{2}}\sin\delta_{2}\\ & 0 & \\ f_{t_{1}}\cos\delta_{1} & + & f_{t_{2}}\cos\delta_{2} \end{array}\right]$$

(13b) $$n_{p}=n_{p_{1}}+n_{p_{2}}=\left[\begin{array}{c} n_{p,x}\\ n_{p,y}\\ n_{p,z} \end{array}\right]=\left[\begin{array}{ccc} -r_{p,y}(f_{t_{1}}\cos\delta_{1} & - & f_{t_{2}}\cos\delta_{2})\\ -r_{p,z}(f_{t_{1}}\sin\delta_{1} & + & f_{t_{2}}\sin\delta_{2})\\ r_{p,y}(f_{t_{1}}\sin\delta_{1} & - & f_{t_{2}}\sin\delta_{2}) \end{array}\right]$$

An approximated result can be obtained by considering that the fins have a small range of movement $\delta_{i}\in \left(-20^{\circ },\;20^{\circ }\right)$, then $\sin\delta_{i}\approx \delta_{i}$ y $\cos\delta_{i}\approx 1$, which has an approximate error of 2%. Using these considerations, the IAM is calculated as shown in Equation (14):

(14a) $$\begin{array}{cc} f_{t_{1,2}} & =\frac{r_{p,y}f_{p,z}\mp n_{p,x}}{2r_{p,y}} \end{array}$$

(14b) $$\begin{array}{cc} \delta_{1,2} & =\frac{-r_{p,y}n_{p,y}\pm r_{p,z}n_{p,z}}{2r_{p,y}r_{p,z}f_{t_{1,2}}} \end{array}$$

Note that $\delta_{1,2}$ is not defined when $f_{t_{1,2}}=0$; this is evident because when there is no flow through the fin, the fin angle does not affect the actuation. When this condition is detected, the reference for $\delta_{1,2}$ is kept in its last known state using a memory block.

**Figure 7:**
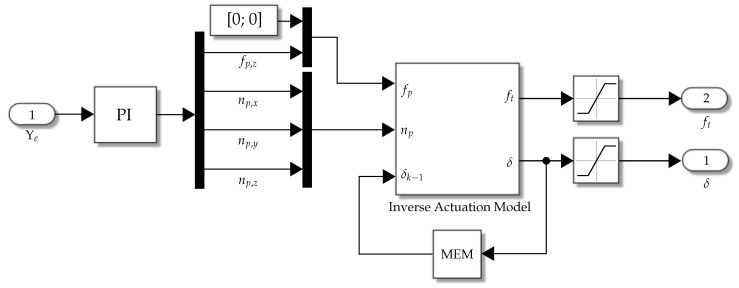
Numerical implementation of the control strategy.

Figure 7 shows the implementation of the control block; note that saturation blocks are added after actuator signals according to the physical limitation of the propellers and fins.

## 4. Results

**Figure 8:**
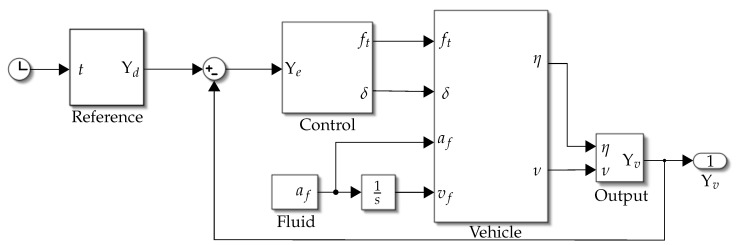
Blocks diagram of the simulator designed in Matlab R2023b and Simulink.

In this section, simulation and experimental results are presented; the complete control loop is presented in the blocks diagram shown in Figure 8, where the implementation of the *vehicle* block is shown in Figure 6 for the simulation and the implementation of the *control* block is shown in Figure 7.

### 4.1. Simulation Results

The simulation results were obtained using Matlab R2023b, Simulink and the VR toolbox for visualization. The selected ODE solver was the fourth-order Runge–Kutta (ODE4), with fixed time step $t_{s}=0.001$ s. Simulation results are independent of the chosen step time; this was verified by running simulations using different values in the range $t_{s}\in \left(0.001,0.1\right)$, where the maximum difference in position was 0.1065% and the maximum difference in orientation was 2.7184%.

The mass and propulsion parameters of the vehicle used for simulation are shown in Table 1; the hydrodynamic parameters of the vehicle are shown in Table 2, they were obtained using Flow Simulation plugin of the SolidWorks 2023 software.

The saturation for the thruster’s force is 20 N and the saturation for the fin’s angle is π/4 rad. The fluid is considered at rest when $a_{fl}=0$. The initial conditions ν(0) and $\eta_{q}\left(0\right)$ are configured in the integration blocks shown in Figure 6.

The gain matrices introduced in Equation (12) are defined as shown in Equation (15).

(15) $$k_{p}=\left[\begin{array}{cccc} 100 & 0 & 0 & 0\\ 0 & 15 & 0 & 0\\ 0 & 0 & 10 & 0\\ 0 & 0 & 0 & 20 \end{array}\right]\;\;\;\;\;\;\;\;\;\;k_{i}=\left[\begin{array}{cccc} 30 & 0 & 0 & 0\\ 0 & 1 & 0 & 0\\ 0 & 0 & 0.5 & 0\\ 0 & 0 & 0 & 5 \end{array}\right]$$

The capabilities of the proposed architecture are tested using four reference trajectories, which exhibit the maneuverability of the vehicle; the references are defined using time and pose keypoints, with a linear interpolator to calculate the intermediate positions.

#### 4.1.1. Test 1: Vertical Submerging and Emerging

The trajectory defined in Table 3 is designed to demonstrate that the vehicle is able to change its navigation style from hover to gliding, in the same way as vertical take off and landing (VTOL) flying drones [39].

The initial conditions for this test are defined by setting the vehicle at rest and located at the surface of the water, vertically, i.e., $\nu \left(0\right)={\left[\begin{array}{cccccc} 0 & 0 & 0 & 0 & 0 & 0 \end{array}\right]}^{⊺}$ and $\eta_{q}\left(0\right)={\left[\begin{array}{ccccccc} 0 & 0 & 0 & 1 & 0 & 0 & 0 \end{array}\right]}^{⊺}$.

**Figure 9:**
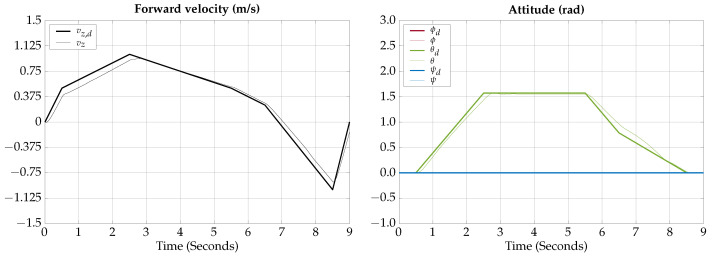
Vertical submerging and emerging test tracking results.

The tracking results for the vertical submerging and emerging test are shown in Figure 9, note that $\phi_{d}=0$ and $\psi_{d}=0$ because in the vertical submerging and emerging test only a trajectory for $\theta_{d}$ is needed.

**Figure 10:**
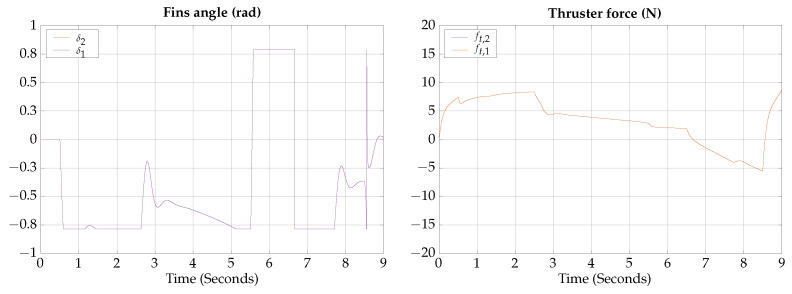
Vertical submerging and emerging test control signals.

The control signals for the vertical submerging and emerging test are shown in Figure 10, note that in the vertical submerging and emerging test the control signals for the fins and thrusters are the same, for the left and right sides, in the absence of perturbations.

**Figure 11:**
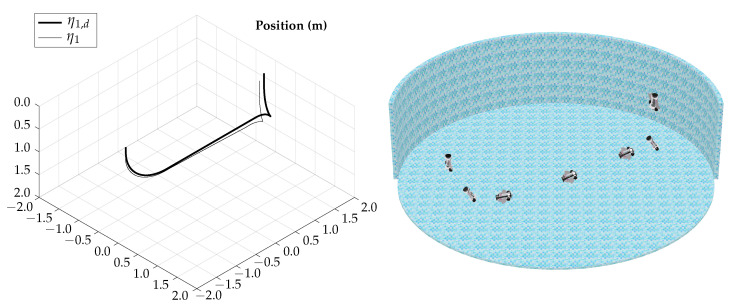
Vertical submerging and emerging test visualization.

The total simulation time is 9 s; the corresponding trajectory of the vertical submerging and emerging test is shown in Figure 11.

According to the simulation results of this test, the vehicle is capable of changing navigation modes within 2 s; however, the fin’s angle reaches the saturation value, indicating that the transition cannot be achieved faster.

#### 4.1.2. Test 2: Horizontal Gliding

The trajectory defined in Table 4 is designed to demonstrate that the vehicle is able to control its lateral movement in gliding mode.

The initial conditions for this test are defined by setting the vehicle at rest at 1 m below the surface of the water, horizontally, i.e., $\nu \left(0\right)={\left[\begin{array}{cccccc} 0 & 0 & 0 & 0 & 0 & 0 \end{array}\right]}^{⊺}$ and $\eta_{q}\left(0\right)={\left[\begin{array}{ccccccc} 0 & 0 & 1 & 0.7071 & 0 & 0.7071 & 0 \end{array}\right]}^{⊺}$.

**Figure 12:**
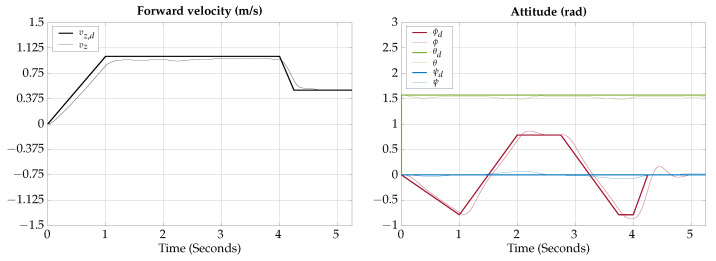
Horizontal gliding test tracking results.

The tracking results for the horizontal gliding test are shown in Figure 12.

**Figure 13:**
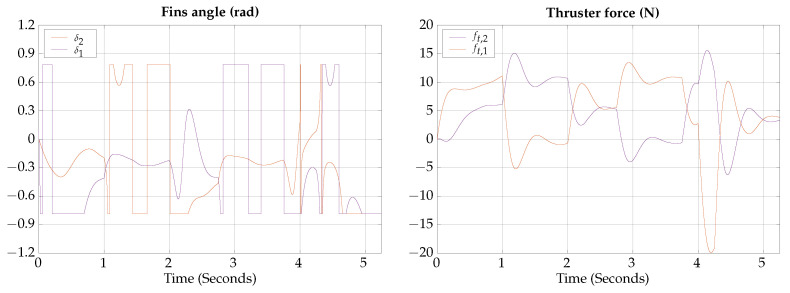
Horizontal gliding test control signals.

The control signals for the horizontal gliding test are shown in Figure 13.

**Figure 14:**
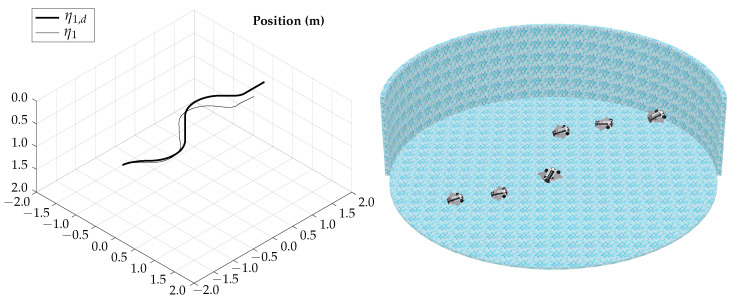
Horizontal gliding test visualization.

The total simulation time is 5.25 s; the corresponding trajectory of the horizontal gliding test is shown in Figure 14.

According to the simulation results of this test, the vehicle is capable of lateral movement when navigating in gliding mode; the actuation configuration allows the vehicle to control heading while maintaining the horizontal orientation.

#### 4.1.3. Test 3: Vertical Gliding

The trajectory defined in Table 5 is designed to demonstrate that the vehicle is able to change depth in gliding mode.

The initial conditions for this test are defined by setting the vehicle at rest at 1 m below the surface of the water, horizontally, i.e., $\nu \left(0\right)={\left[\begin{array}{cccccc} 0 & 0 & 0 & 0 & 0 & 0 \end{array}\right]}^{⊺}$ and $\eta_{q}\left(0\right)={\left[\begin{array}{ccccccc} 0 & 0 & 1 & 0.7071 & 0 & 0.7071 & 0 \end{array}\right]}^{⊺}$.

**Figure 15:**
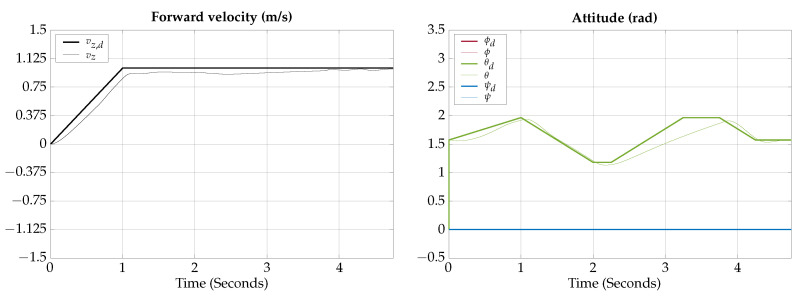
Vertical gliding test tracking results.

The tracking results for the vertical gliding test are shown in Figure 15, note that $\phi_{d}=0$ and $\psi_{d}=0$ because in the vertical gliding test only a trajectory for $\theta_{d}$ is needed.

**Figure 16:**
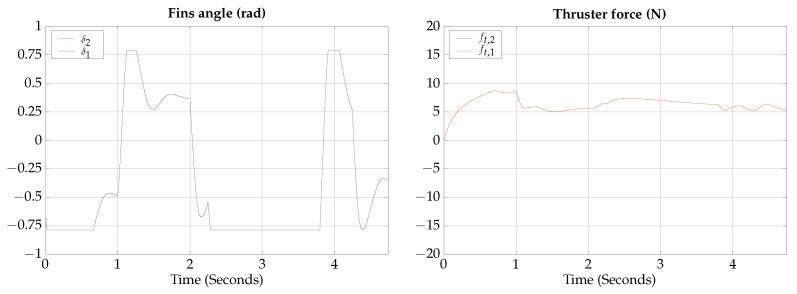
Vertical gliding test control signals.

The control signals for the vertical gliding test are shown in Figure 16, note that in the vertical gliding test the control signals for the fins and thrusters are the same, for the left and right sides, in the absence of perturbations.

**Figure 17:**
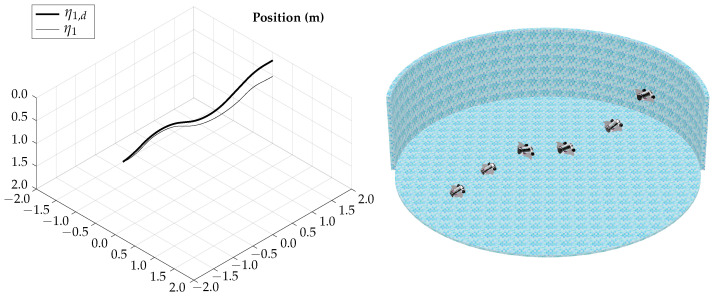
Vertical gliding test visualization.

The total simulation time is 4.75 s; the corresponding trajectory for the vertical gliding test is shown in Figure 17.

According to the simulation results of this test, the vehicle is capable of controlling its depth when navigating on gliding mode; the actuation configuration allows the vehicle to change depth by following a reference in orientation.

#### 4.1.4. Test 4: Horizontal Axial Roll

The trajectory defined in Table 6 is designed to demonstrate that the vehicle is able to control its lateral inclination in gliding mode.

The initial conditions for this test are defined by setting the vehicle at rest at 1 m below the surface of the water, horizontally, i.e., $\nu \left(0\right)={\left[\begin{array}{cccccc} 0 & 0 & 0 & 0 & 0 & 0 \end{array}\right]}^{⊺}$ and $\eta_{q}\left(0\right)={\left[\begin{array}{ccccccc} 0 & 0 & 1 & 0.7071 & 0 & 0.7071 & 0 \end{array}\right]}^{⊺}$.

**Figure 18:**
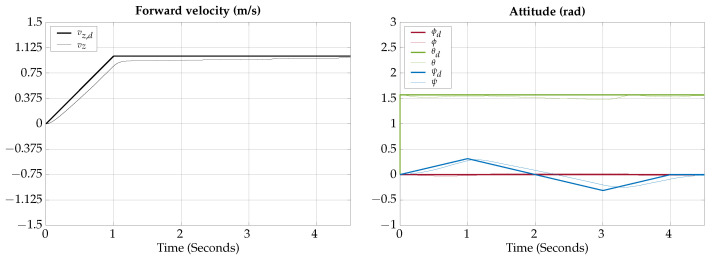
Horizontal axial roll test tracking results.

The tracking results for the horizontal axial roll test are shown in Figure 18.

**Figure 19:**
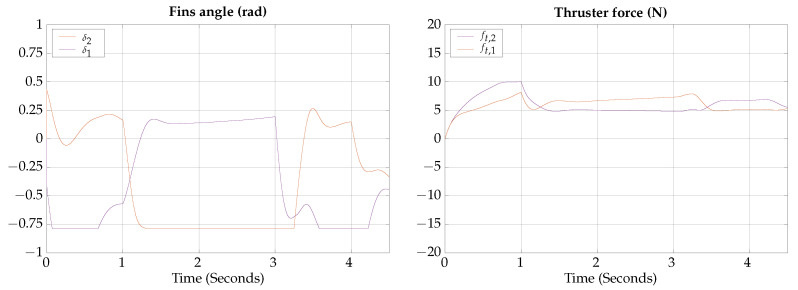
Horizontal axial roll test control signals.

The control signals for the horizontal axial roll test are shown in Figure 19.

**Figure 20:**
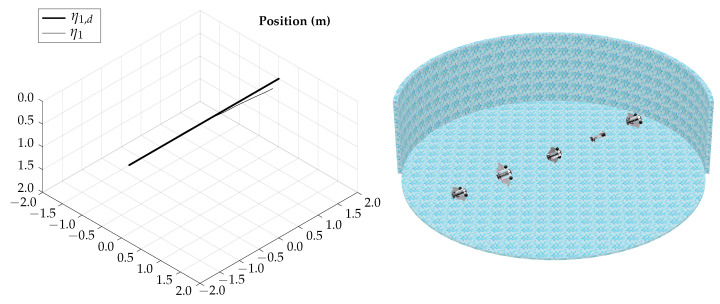
Horizontal axial roll test visualization.

The total simulation time is 4.5 s; the corresponding trajectory for the horizontal axial roll test is shown in Figure 20.

According to the simulation results of this test, the vehicle is capable of controlling the lateral orientation when navigating on gliding mode, the actuation configuration allows the vehicle to rotate along the axis movement to compensate for possible perturbations.

### 4.2. Experimental Results

The experimental tests were carried out in a 4.27 m diameter pool filled with tap water; communication with the ROV was implemented through an ethernet cable and control signals were user-defined using a commercial joystick; the control and orientation signals were recorded using ROS middleware.

#### 4.2.1. Test 1: Vertical Submerging and Emerging

This test is designed to demonstrate, using experimental results, that the proposed platform is able to change its navigation style from hover to gliding via teleoperation commands defined by a user.

**Figure 21:**
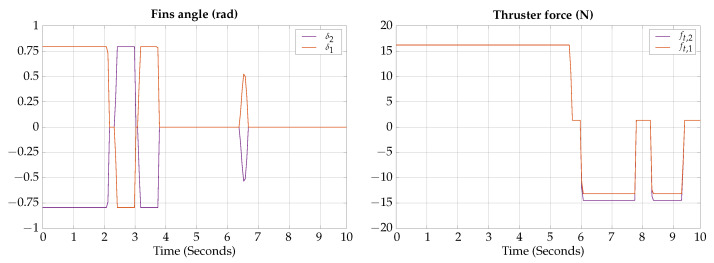
Vertical submerging and emerging experimental test control signals.

The control signals for the vertical submerging and emerging experimental test are presented in Figure 21.

**Figure 22:**
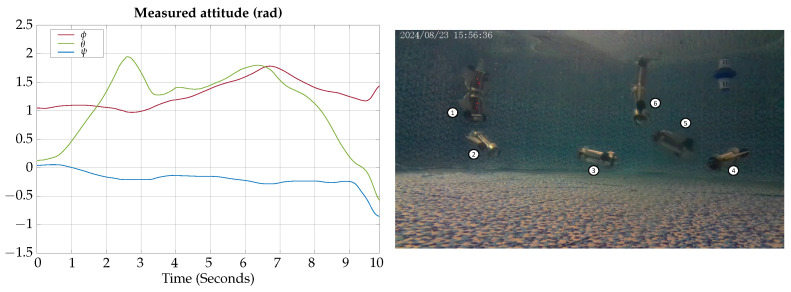
Vertical submerging and emerging experimental test attitude signals. The circled numbers represent the sequence of the movement.

The measured angles for the attitude of the robot during the vertical submerging and emerging experimental test are shown in Figure 22; also, a photographic sequence is shown where the experimental trajectory of the robot can be observed.

According to the experimental results of this test, the vehicle can be teleoperated for changing navigation modes; the results are similar to the ones obtained by simulation, confirming that the vehicle can change from hover to gliding mode in about 2 s.

#### 4.2.2. Test 2: Horizontal Gliding

This test is designed to demonstrate, using experimental results, that the proposed platform is able to control its lateral movement in gliding mode via teleoperation commands defined by a user.

**Figure 23:**
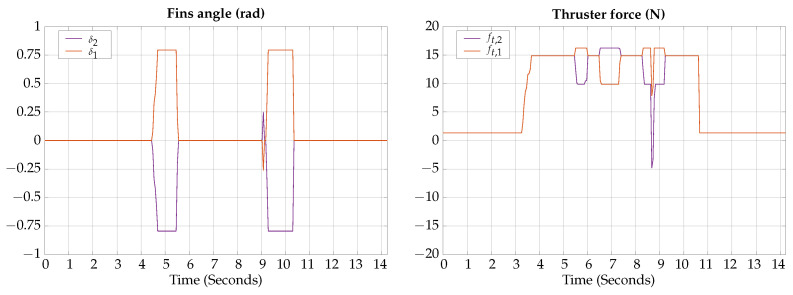
Horizontal gliding experimental test control signals.

The control signals for the vertical gliding experimental test are presented in Figure 23.

**Figure 24:**
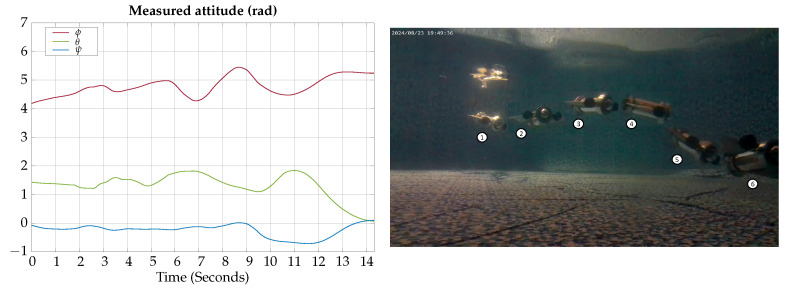
Horizontal gliding experimental test attitude signals. The circled numbers represent the sequence of the movement.

The measured angles for the attitude of the robot during the horizontal gliding experimental test are shown in Figure 24; also, a photographic sequence is shown where the experimental trajectory of the robot can be observed.

According to the experimental results of this test, the vehicle can be teleoperated to change lateral heading when moving in gliding mode; however, it requires practice to correctly teleoperate the horizontal and vertical motion simultaneously.

#### 4.2.3. Test 3: Vertical Gliding

This test is designed to demonstrate, using experimental results, that the proposed platform is able to change depth in gliding mode via teleoperation commands defined by a user.

**Figure 25:**
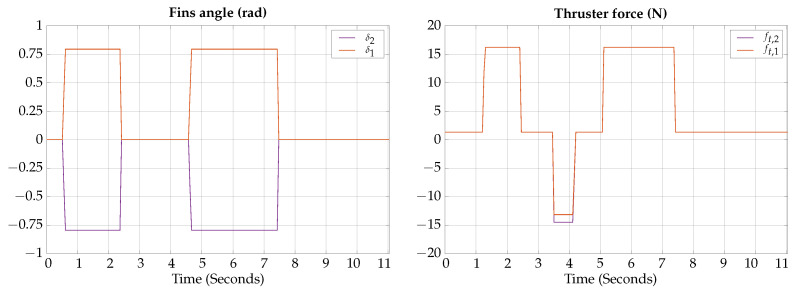
Vertical gliding experimental test control signals.

The control signals for the vertical gliding experimental test are presented in Figure 25.

**Figure 26:**
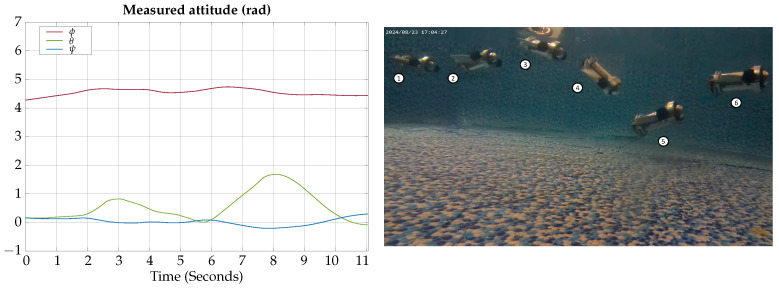
Vertical gliding experimental test attitude signals. The circled numbers represent the sequence of the movement.

The measured angles for the attitude of the robot during the vertical gliding experimental test are shown in Figure 26; also, a photographic sequence is shown where the experimental trajectory of the robot can be observed.

According to the experimental results of this test, the vehicle can be teleoperated to change depth when moving in glide mode; however, due to the location of the center of buoyancy relative to the center of gravity, it requires less torque to move downwards than upwards, so the movement is not symmetrical.

## 5. Discussion

The simulation results show that the proposed vehicle can be teleoperated in gliding mode to follow reference orientations, as presented in Figure 9, Figure 12, Figure 15, and Figure 18; however, the position of the vehicle may differ from the expected trajectory if the delay time of the transient respond is not short enough or if the actuators reach saturation values, as shown in Figure 11, Figure 14, Figure 17, and Figure 20.

The experimental results, presented in Figure 22, Figure 24, and Figure 26, show that the platform can be challenging to teleoperate in a reduced space; one reason is that the degrees of freedom of the vehicle are highly coupled, as can be seen in Equations (13) and (14), so it is necessary to implement full pose control so the teleoperation process becomes more suitable for real-world scenarios.

**Figure 27:**
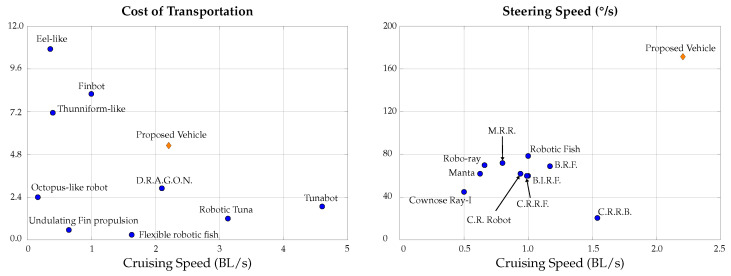
Comparison of cost of transportation and steering speed of different bio-inspired underwater vehicles: C.R.R.B. [15], Manta [16], Thunniform-like [22], M.R.R. [26], Octopus-like robot [40], Eel-like [41], Undulating Fin propulsion [42], Finbot [43], Flexible robotic fish [44], D.R.A.G.O.N. [45], Robotic Tuna [46], Tunabot [47], Cownose Ray-I [48], Robo-ray [49], C.R. Robot [50], Robotic Fish [51], C.R.R.F. [52], B.I.R.F. [53] and B.R.F. [54].

Based on the simulation and experimental results, it is concluded that the proposed bio-inspired design is highly efficient and maneuverable, suitable for inspection and mapping applications. Figure 27 shows a comparison of different bio-inspired vehicles considering cost of transportation and steering speed against cruising speed. According to this comparison, the proposed prototype has a competitive cost of transportation when compared to vehicles inspired by different marine creatures, with an outstanding maneuverability, as seen in the comparison of steering speed.

Although flapping is a highly efficient form of propulsion, it provides poor maneuverability compared with the proposed hybrid design that combines thrusters and fins.

The vehicle performance can be improved by reducing drag during glide mode; this can be achieved by modifying the design of the electronic capsule; a reduction in drag will increase the cruising speed, which in turn reduces the transport cost. The steering speed can also be improved by increasing the area of the mobile section of the fins and/or adding another joint for better fluid redirection.

In addition to the mechanical improvements that can be implemented on the platform, there is plenty of work that can be performed regarding the automatic control and localization strategies that can be designed using the proposed mathematical model.

In conclusion, the proposed underwater ROV design, based on traditional thrusters and bio-inspired articulated fins, is an efficient platform capable of vertical submerging and emerging, is an energy efficient vehicle compared with similar projects, and exhibits outstanding maneuverability. The proposed architecture, considered as an experimental control platform, is a challenging system for the design of automatic controllers but also attractive for real-world applications such as exploration and mapping.

## Figures and Tables

**Table 1:** Mass and propulsion parameters of the vehicle.

$m_{v}$	$I_{g_{v}}$	$r_{p_{1}/v}^{v}$	$r_{p_{2}/v}^{v}$
(kg)	(kg m^2^)	(m)	(m)
3.956	$\left[\begin{array}{ccc} 0.063 & 0 & 0\\ 0 & 0.045 & 0\\ 0 & 0 & 0.026 \end{array}\right]$	$\left[\begin{array}{c} 0\\ -0.15\\ -0.084 \end{array}\right]$	$\left[\begin{array}{c} 0\\ 0.15\\ -0.084 \end{array}\right]$

**Table 2:** Hydrodynamic parameters of the vehicle.

$m_{f_{v}}$	$I_{f_{v}}$	$r_{b_{v}/v}^{v}$	$d_{v}$	$D_{v}$
(kg)	(kg m^2^)	(m)	(Ns/m)	(Nms/rad)
4.3	$\left[\begin{array}{ccc} 0.055 & 0 & 0\\ 0 & 0.038 & 0\\ 0 & 0 & 0.023 \end{array}\right]$	$\left[\begin{array}{c} 0\\ 0\\ -0.009 \end{array}\right]$	$\left[\begin{array}{ccc} 100 & 0 & 0\\ 0 & 10 & 0\\ 0 & 0 & 10 \end{array}\right]$	$\left[\begin{array}{ccc} 1 & 0 & 0\\ 0 & 0.9 & 0\\ 0 & 0 & 5 \end{array}\right]$

**Table 3:** Vertical submerging and emerging test reference table.

*T*	$v_{d,z}$	$\phi_{d}$	$\theta_{d}$	$\psi_{d}$
(s)	(m/s)	(rad)	(rad)	(rad)
0	0	0	0	0
0.5	0.5	0	0	0
2	1	0	π/2	0
3	0.5	0	π/2	0
1	0.25	0	π/4	0
2	−1	0	0	0
0.5	0	0	0	0

**Table 4:** Horizontal gliding test reference table.

*T*	$v_{d,z}$	$\phi_{d}$	$\theta_{d}$	$\psi_{d}$
(s)	(m/s)	(rad)	(rad)	(rad)
0	0	0	π/2	0
1	1	−π/4	π/2	0
1	1	π/4	π/2	0
0.75	1	π/4	π/2	0
1	1	−π/4	π/2	0
0.25	1	−π/4	π/2	0
0.25	0.5	0	π/2	0
1	0.5	0	π/2	0

**Table 5:** Vertical gliding test reference table.

*T*	$v_{d,z}$	$\phi_{d}$	$\theta_{d}$	$\psi_{d}$
(s)	(m/s)	(rad)	(rad)	(rad)
0	0	0	π/2	0
1	1	0	5π/8	0
1	1	0	3π/8	0
0.25	1	0	3π/8	0
1	1	0	5π/8	0
0.5	1	0	5π/2	0
0.5	1	0	π/2	0
0.5	1	0	π/2	0

**Table 6:** Horizontal axial roll test reference table.

*T*	$v_{d,z}$	$\phi_{d}$	$\theta_{d}$	$\psi_{d}$
(s)	(m/s)	(rad)	(rad)	(rad)
0	0	0	π/2	0
1	1	0	π/2	π/10
2	1	0	π/2	−π/10
1	1	0	π/2	0
0.5	1	0	π/2	0

## Data Availability

Data are contained within the article.
